# Platelet-rich plasma respectively reduces and promotes adipogenic and myofibroblastic differentiation of human adipose-derived stromal cells via the TGFβ signalling pathway

**DOI:** 10.1038/s41598-017-03113-0

**Published:** 2017-06-07

**Authors:** Bérengère Chignon-Sicard, Magali Kouidhi, Xi Yao, Audrey Delerue-Audegond, Phi Villageois, Pascal Peraldi, Patricia Ferrari, Yves Rival, David Piwnica, Jérôme Aubert, Christian Dani

**Affiliations:** 1 Université Côte d’Azur, CNRS, Inserm, iBV, Faculté de Médecine, 06107 Nice Cedex 2, France; 2 Plastic, Reconstructive and Hand Surgery Department, Hôpital Pasteur 2, Nice, France; 3 Research Galderma, Sophia, Antipolis France; 4 Biochemistry Department, Hôpital Pasteur 1, Nice, France

## Abstract

Autologous fat grafting is a gold standard therapy for soft tissue defects, but is hampered by unpredictable postoperative outcomes. Fat graft enrichment with adipose-derived stromal cell (ASCs) was recently reported to enhance graft survival. Platelet-rich plasma (PRP) has also emerged as a biologic scaffold that promotes fat graft viability. Combined ASC/PRP fat grafting enrichment is thus a promising new regenerative medicine approach. The effects of PRP on ASC proliferation are well documented, but the impact of PRP on ASC differentiation has yet to be investigated in depth to further elucidate the PRP clinical effects. Here we analyzed the human ASC fate upon PRP treatment. PRP was found to sharply reduce the potential of ASCs to undergo differentiation into adipocytes. Interestingly, the PRP anti-adipogenic effect was accompanied by the generation of myofibroblast-like cells. Among the various factors released from PRP, TGFβ pathway activators played a critical role in both the anti-adipogenic and pro-myofibroblastic PRP effects. Overall, these data suggest that PRP participates in maintaining a pool of ASCs and in the repair process by promoting ASC differentiation into myofibroblast-like cells. TGFβ may provide an important target pathway to improve PRP clinical outcomes.

## Introduction

Autologous fat grafting is a gold standard therapy for soft tissue defects, correction or augmentation in reconstructive and plastic surgery. Autologous fat grafting is particularly useful after tumor removal, in breast reconstruction surgery after mastectomy, as well as in repairing extensive facial deformities caused by injury, illness or congenital abnormalities. This treatment capitalizes on the large quantities of autologous fat that are easy to obtain in various parts of the body with minimum morbidity for patients. However, fat grafting is still controversial due to the postoperative outcome predictability. The main drawbacks of this technique are the variable engraftment results, resorption and cyst formation due to fat necrosis. Platelet-rich plasma (PRP) has recently emerged as an autologous scaffold able to promote neovascularization and fat graft viability^1, 2^. Enrichment with adipose-derived stromal/stem cells (ASCs) has also been shown to enhance fat graft survival, likely through both ASC differentiation and secretion of angiogenic factors^3^. PRP has also been reported to stimulate ASCs secretion of angiogenic factors^4, 5^. Co-transplantation of ASCs and PRP is thus a promising cell therapy approach in regenerative medicine.

PRP contains a variety of growth factors, such as platelet-derived growth factors (PDGFs) and vascular endothelial growth factors (VEGFs), with neovascularization properties. PRP also releases factors from members of the transforming growth factor-β (TGF β) family that were shown to be potent anti-adipogenic factors and key mediators in the conversion of human ASCs into myofibroblast-like cells^6, 7^. The functional role of TGFβ in PRP effects has, however, not been demonstrated. Myofibrobasts, characterized by alpha-smooth actin (αSMA) expression and collagen secretion, are key partners in the tissue repair process. However, as excessive collagen deposition is associated with fibrotic lesions, we considered it essential to study the TGFβ pathway with regard to PRP effects on ASC adipogenic and myofibroblastic differentiation.

In this study, we investigated the effects of individual PRPs prepared from six healthy donors on the differentiation of three different ASC sources (see Materials and Methods). We show that the ASC adipocyte differentiation potential was sharply reduced in the presence of PRP. The PRP anti-adipogenic effect was accompanied by an increase in alpha smooth muscle actin expressing cells and type 1 collagen secretion, the hallmark of myofibroblast-like cells. TGF pathway activators released from PRP were found to have a critical role in this phenomenon as the small SB431542 molecule, i.e. a selective TGFβ pathway inhibitor, abolished both the anti-adipogenic and pro-myofibroblastic effects of PRP.

## Results

### PRP reduced adipogenic differentiation of ASCs

**Figure 1:**
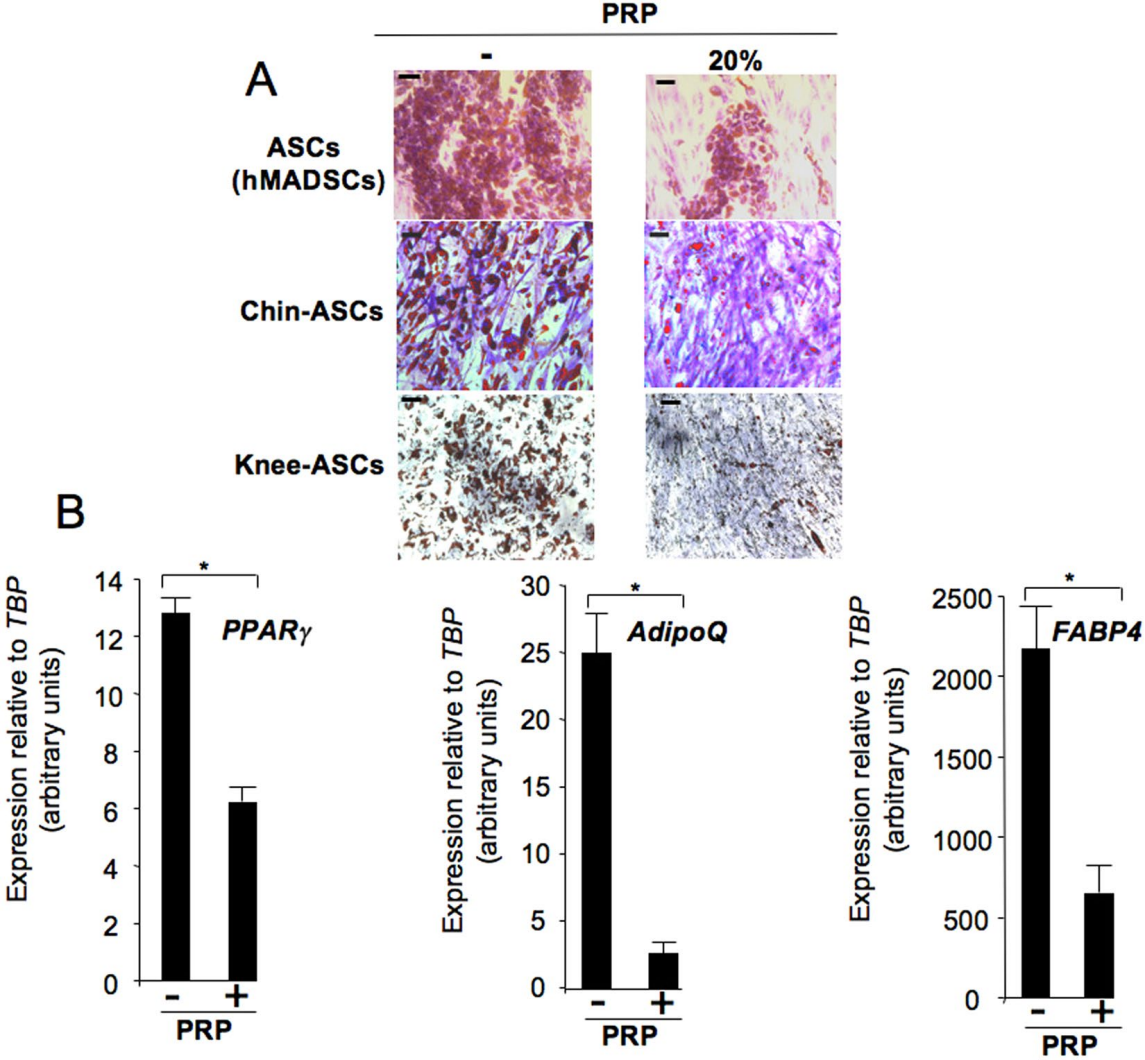
PRP inhibited ASC adipocyte differentiation. (**A**) ASCs (hMADScs), chin- and knee-ASC primary cultures were induced to undergo adipocyte differentiation in the absence (−) or presence (+) of 20% PRP. After 10 days, cells were fixed and stained with Oil Red O to visualize lipid droplets, then with nonspecific violet crystals to stain the cell layer. Bar scale: 50 μm. (**B**) ASCs were maintained as in (**A**) and RNAs were prepared to analyse expression of the indicated genes by real-time PCR. Values are means ± SEM (n = 3 individual PRPs). *p < 0.05.

PRPs obtained from six healthy subjects were first tested for their ability to promote ASC proliferation. ASCs derived from paired chin- and knee-fat depots^8^ were maintained in the presence of 20% PRP. In agreement with the findings of previous studies^4, 5, 9–11^, the addition of PRP dramatically promoted ASC proliferation (Fig. S1). As the impact of PRP treatment on the ASC fate has yet to be investigated, we analyzed PRP effects on the ASC adipogenic potential. The ratio of PRP used in previous publications is variable. Liao *et al*.^9^ showed that PRP displays an effect on differentiation from 5% to 20%, whereas in a clinical study, Cervelli *et al*.^11^ showed that 40% PRP was optimal for graft maintenance. We show that the six individually tested PRPs dramatically inhibited knee-ASC adipocyte formation when used at 20%, as revealed by the lower number of adipocytes and lipid contents quantified in the PRP-treated cultures (Fig. S2). Therefore, 20% PRP was used for subsequent experiments. PRPs could have an effect when used at lower concentrations, as suggested by the effects observed when two PRPs were tested at 5% and 10% (Fig. S2). The effects of low PRP concentrations on ASC fate remain to be investigated in more details. The anti-adipogenic effect of 20% PRP was reproduced on three different ASC sources (Fig. 1A). Finally, the effect was confirmed at the molecular level, as shown by the lower expression of adipogenic genes such as *PPARγ* (an adipogenic master gene), *AdipoQ* (an adipocyte-specific adipokine coding for adiponectin) and *FABP4* (a gene coding for a fatty acid carrier specifically expressed during adipocyte differentiation) in PRP-treated cells (Fig. 1B).

### TGFβ signalling pathway inhibitor SB431542 reversed PRP anti-adipogenic effects

**Figure 2:**
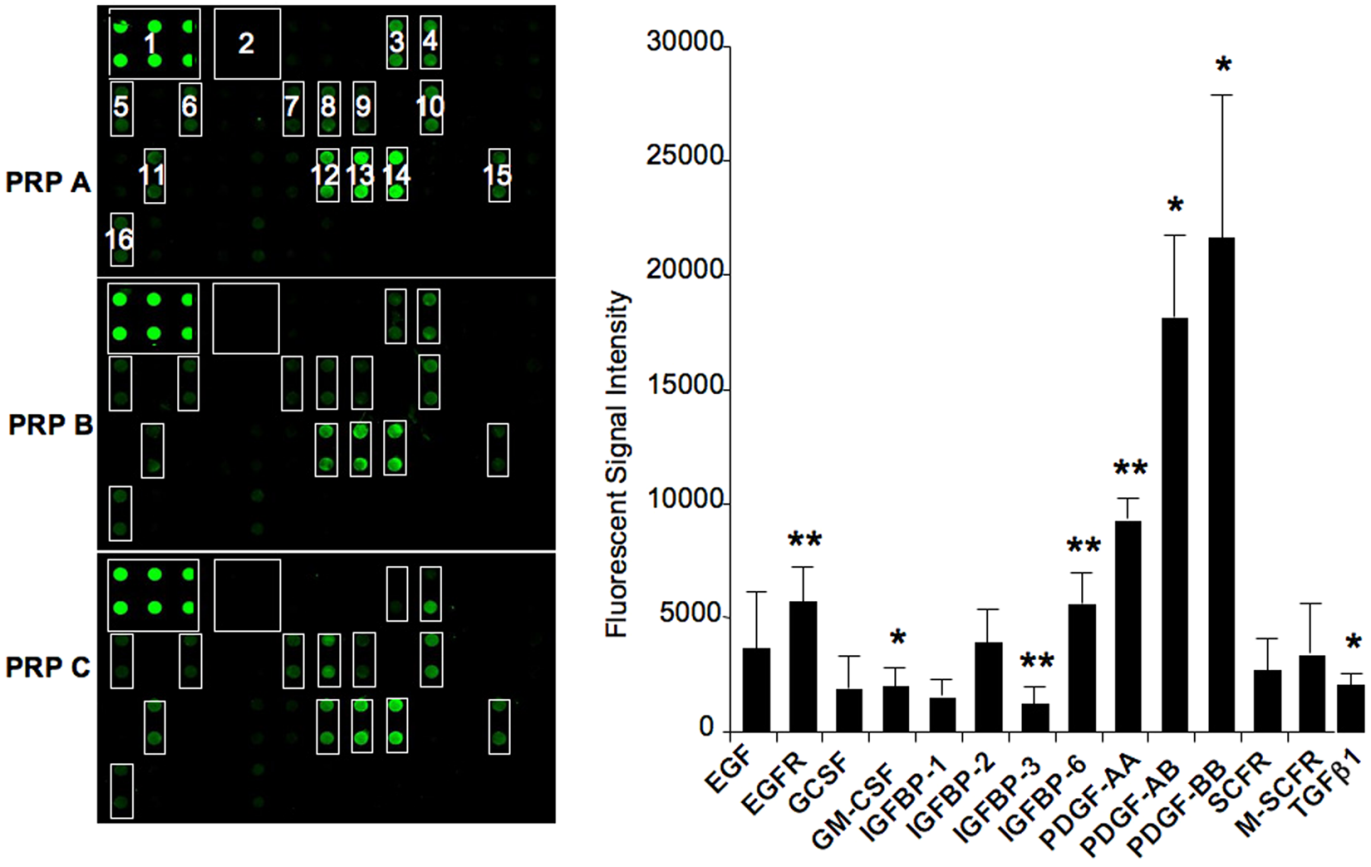
PRP profiling. Cytokines released from PRPs were detected using the RayBio C-Series Human Growth factor Antibody kit. Left panel: Images of arrays probed with indicated PRPs. Cytokines are spotted in duplicate and those released from PRP appear as green dots. The positions and names of cytokines that generated a detectable signal are as follows. 1: Positive control; 2: Negative control; 3: EGF; 4: EGFR; 5: GCSF; 6: GM-CSF; 7: IGFBP-1; 8: IGFBP-2; 9: IGFBP-3; 10: IGFBP-6; 11: M-CSFR; 12: PDGF-AA; 13: PDGF-AB; 14: PDGF-BB; 15: SCFR; 16: TGFb1. Cytokines that were present on the array and non detectable are as follows: amphiregulin (AR, AREG), bFGF, b-NGF, FGF-4, FGF-6, FGF-7, GDNF, HB-EGF, HGF, IGFBP-4, IGF-I, IGF-I R, IGF-II, M-CSF, NT-3, NT-4, PDGF R a, PDGF R b, PLGF, SCF, TGF alpha, TGF beta2, TGF beta3,VEGF-A,VEGFR2,VEGFR3,VEGF-D. Right panel: factors/cytokines presenting a detectable fluorescent signal are indicated. Values are the fluorescence means ± SEM (n = 3 individual PRPs.) *p < 0.05; **p < 0.01.

**Figure 3:**
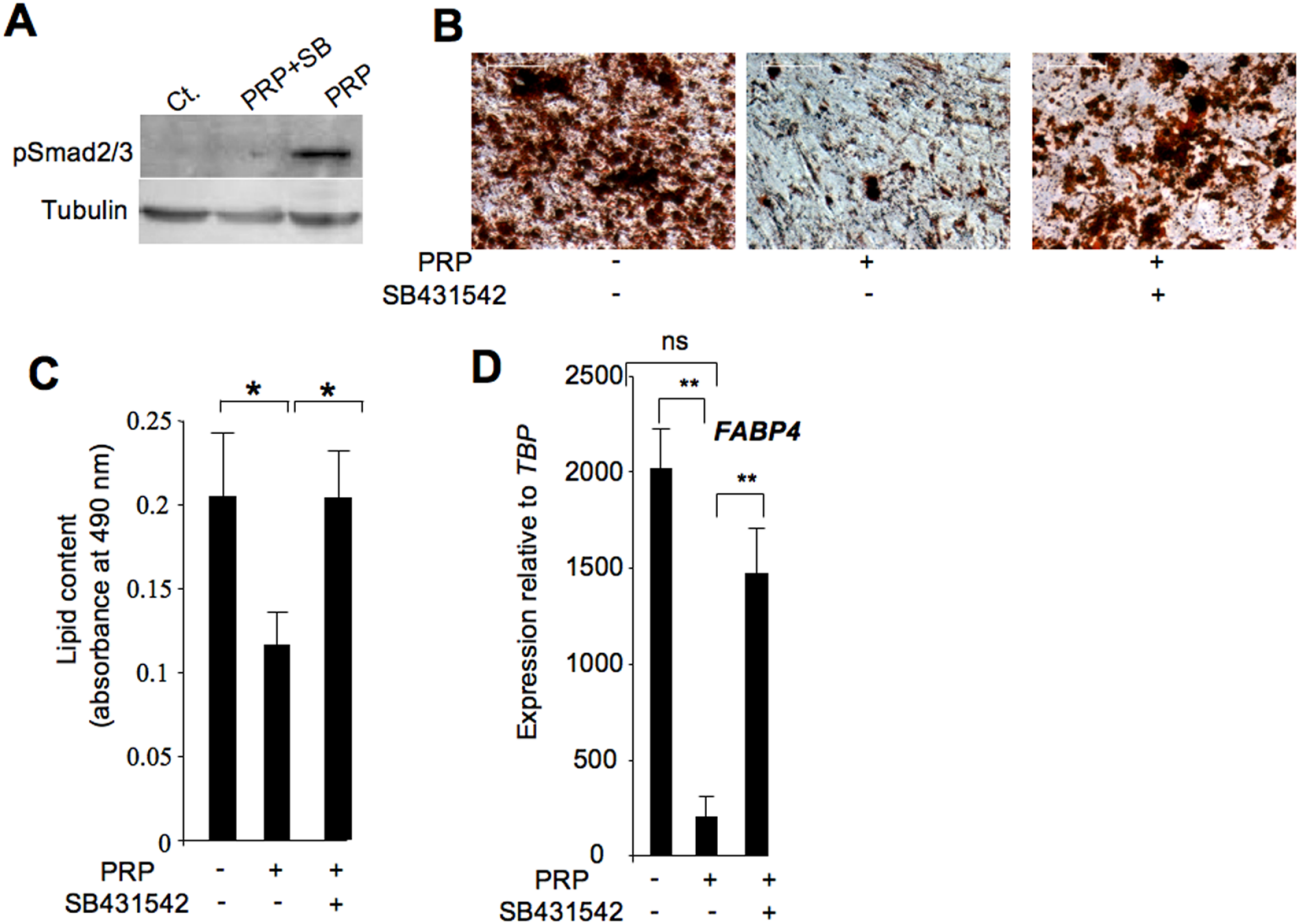
TGFβ pathway inhibitor SB 431542 reversed anti-adipogenic PRP effects. (**A**) ASC (hMADSCs) proteins were extracted 1 hour after treatment with 20% PRPs in the absence or presence of 5 μM SB431542 and analyzed for expression of total Smad2/3 and activated phospho Smad2/3. The blots were cropped. Full-length blots are presented in Supplementary Fig. S5. (**B**) A representative photomicrophographic record of ASCs (hMADSCs) treated or not with 20% PRP and 5 μM SB431542. Bar scale: 50 μm. (**C**) The lipid content was measured by quantification of Oil Red O staining. Values are the mean ± SEM (n = 4) for individual PRPs. (**D**) ASCs (hMADScs) and chin-ASCs were induced to undergo adipocyte differentiation in the presence of four individual PRPs. After 10 days, RNAs were prepared and *FABP4* adipogenic gene expression was analyzed by real-time PCR. Values are the mean ± SEM (n = 4 for individual PRPs). **p < 0.01.

We profiled PRPs as the first step in identifying molecular mechanisms responsible for the PRP anti-adipogenic effect. An antibody array containing cytokines/growth factors, some of which are known to play a role in adipogenesis, was thus screened. Of the 40 factors in the array (see Methods), eight displayed a fluorescent signal significantly above the background, e.g. EGFR, GM-CSF, IGFBP3, IGFBP6, PDGF-AA, PDGF-AB, PDGF-BB and TGFβ1 (Fig. 2). ELISA assays confirmed that IGFBP3, PDGF-AA, leptin (not spotted in the array) and TGFβ1 were released from PRPs (Table S1). We focused on the potential role of TGFβ1 in the PRP anti-adipogenic effect. The TGFβ pathway was a strong candidate for involvement in the PRP effect because this pathway was previously reported to have a potent anti-adipogenic effect mediated by Smad2/3 phosphorylation^7, 12^. As expected, ASC adipocyte differentiation was inhibited in the presence of 2 ng/ml TGFβ1 and the TGFβ1 anti-adipogenic effect was reversed using 5 μM SB431542, a selective TGFβ pathway inhibitor^13^ (Fig. S4). This SB431542 concentration was sufficient to inhibit TGFβ1-induced Smad 2/3 activation (Fig. S4). However, the functional role of TGFβ in the PRP mixture has yet to be demonstrated. As shown in Fig. 3A, Smad 2/3 phosphorylation was induced upon PRP treatment and PRP-induced Smad2/3 phosphorylation was reversed in the presence of SB431542. These data strongly suggest that the TGFβ pathway was activated by PRP in ASCs and could play a role in the PRP anti-adipogenic effect. To test this hypothesis, ASCs were induced to undergo adipocyte differentiation in the presence of PRP with or without the SB431542 compound. As shown, the lipid content and *FABP4* adipogenic gene expression were restored when cells were treated with 20% PRP in the presence of 5 μM SB431542 (Fig. 3B–D). Together, these findings supported the hypothesis that the TGFβ pathway activators released from PRP are major mediators of the PRP anti-adipogenic effect.

### PRP induced myofibroblast-like cell phenotype reversed by SB431542

**Figure 4:**
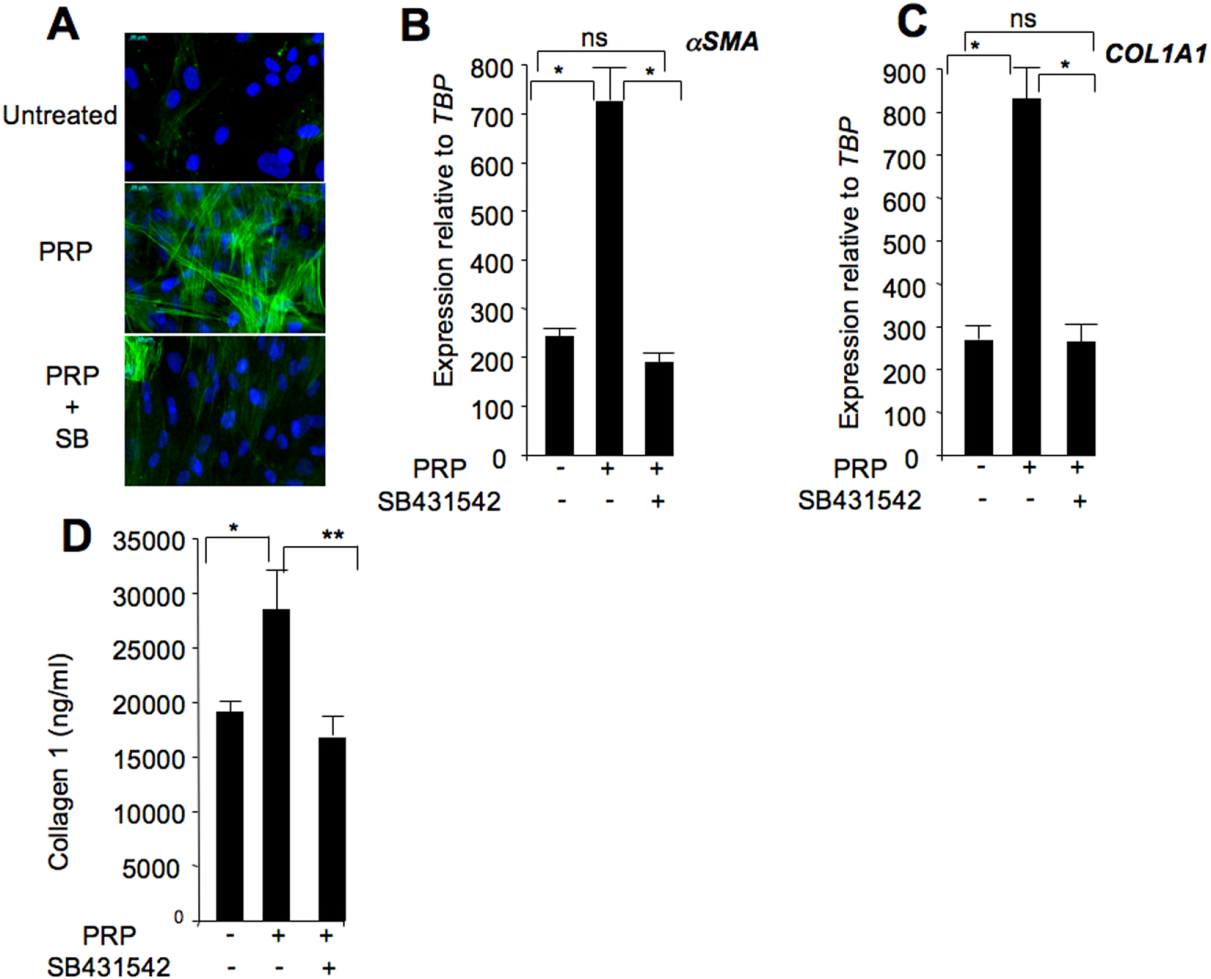
PRP-induced myofibrobast-like phenotype reversed by SB431542. ASCs were induced to undergo adipocyte differentiation for 11 days in the indicated conditions. (**A**) Generation of αSMA-expressing cells was revealed by immunoflorescence. (**B**,**C**) RNAs were prepared and expression of the indicated genes was analyzed by real-time PCR. Values are the mean ± SEM (n = 4 individual PRPs). (**D**) Media were collected and collagen type 1 secretion was quantified. Values are the mean ± SEM (n = 4 individual PRPs). *p < 0.05, **p < 0.01.

The TGFβ pathway is considered to be the master pathway driving myofibroblast generation in physiological and pathological processes^14^. In addition, we previously reported that, in an obesity context, TGFβ1 treatment converts human ASCs into myofibroblast-like cells at the expense of adipocyte differentiation^6^. As expected, ASC myofibroblastic differentiation induced by TGFβ1 was reversed by SB431542 (see Fig. S3). These findings prompted us to investigate the fate of PRP-treated ASCs. ASCs were thus maintained in adipogenic conditions in the absence or presence of PRP and myofibroblastic marker expression was investigated. Interestingly, PRP promoted the generation of αSMA-expressing cells and *alpha-smooth muscle actin* (α*SMA*) gene expression (Fig. 4A,B, also see Fig. S3). The PRP-induced ASC myofibroblastic-like phenotype was confirmed by overexpression of α*1 chain 1 of type 1 collagen* (*COL1A1*) genes, as well as by the secretion of type 1 collagen (Fig. 4C,D). Overall, these data indicated that PRP could convert ASCs into a myofibroblast-like phenotype. As for adipogenesis, SB431542 reversed PRP-induced myofibroblastic differentiation, indicating that the TGFβ pathway plays a critical role in regulation of the PRP-induced ASC fate.

## Discussion

ASC and PRP-supplemented fat grafts are promising for reconstructive surgery. The beneficial effects of PRP include its potential to promote neovascularization and enhance fat graft viability. PRP profiling data indicated that various factors were released from our PRP preparations, as previously reported. PDGFs and TGF β1 release have been previously reported and at concentrations similar to those shown in Table S1 ^2, 15, 16^. Our experiments also revealed factors released from PRPs that were not previously reported, such as IGFBP3, IGFBP6 and leptin. Note that, in addition to being adipocyte differentiation regulators, these latter factors were also described as being critical modulators of angiogenesis^17, 18^. The specific role of these factors has yet to be investigated, but they likely play a role in the clinical effects of PRP. One potential beneficial effect of ASC enrichment is the maintenance of a longer-term graft volume. Our data indicated that PRP promotes ASC proliferation, as previously reported, while also inhibiting ASC adipogenic differentiation. We speculate that this phenomenon observed *ex vivo* could participate in maintaining a pool of undifferentiated ASCs to enable adipocyte renewal when PRP growth factors are resorbed. Here, we showed that PRPs had an effect on the ASCs derived from the pubis fat pad from a 4 month-old child, and on ASCs derived from chin and knee adipose tissues from a 72 year-old female subject. We also observed PRP effects on ASCs derived from chin- and knee- fat depots from two 61 year-old female subjects of and one 46 year-old female subject (not shown). The effects of PRP on other sources of cells used for autologous fat grafting, such as thigh, hip, and abdominal fat pads, have yet to be analyzed, but it is tempting to propose that PRP effects are age- and fat depot-independent.

It was previously reported that mice treated with PRP displayed increased osteogenesis in bone marrow^19^. Interestingly, osteoblast formation was observed at the expense of bone-marrow adipogenesis, in full agreement with the PRP anti-adipogenic effect we observed with human ASCs. PRP anti-adipogenic effects on ASCs were also previously reported^9^. However, the authors of that study did not functionally identify PRP factors mediating this effect, nor did they investigate the fate of PRP-treated ASCs. It is now essential to identify the signalling pathways responsible for PRP effects to enhance clinical applications. We demonstrated, for the first time, that PRP induced ASC myofibrogenesis differentiation at the expense of adipogenesis and that the TGFβ pathway played a critical role in these effects. Pro-adipogenic effects of PRP have also been reported, but only when PRP was associated with high insulin concentrations^11^. At these concentrations it is known that insulin mimics IGF1 effects. Interestingly, IGF-I signals cross-talk at multiple levels with various components of the TGFβ signaling pathway^20^. Further studies on the cross-talk between IGF1 and TGFβ pathways would therefore be of interest, while also analysing their impact on PRP effects.

Finally, the ability of PRP to convert ASCs into myofibroblast-like cells could have clinical consequences. Myofibroblasts are known to participate in tissue repair processes while also being the cellular source of fibrosis when there is excessive collagen deposition. Recently, adipocyte-myofibroblast transition has been proposed as one of the cellular mechanisms leading to cutaneous fibrosis pathogenesis^21^. The PRP-induced ASC myofibroblast-like phenotype would thus require further investigation to assess both the benefits and potential negative outcomes. Our study highlighted a molecular mechanism that might be involved in the clinical effects of PRP when combined with ASC-enriched fat grafting. Note that PRPs can have different properties according to the system used for their purification^15, 16^. The myofibrogenic potential of PRPs from different preparation has yet to be analyzed. The next step would be to investigate our *in vitro* approach in an animal model. *In vivo* analysis of the impact of PRP depleted of TGFβ activity by co-injection of PRP with pharmacological molecule inhibitors of the TGFβ pathway could provide an opportunity to enhance fat grafting.

## Methods

### Isolation, characterization and culture of adipose-derived stem/stromal cells

Experiments were carried out using adipose stem cells (ASCs) derived from the pubic region fat pad of a 4-month old male donor. These cells were previously named hMADS3 cells^22^ and are called ASCs (hMADSCs) here, compliance with the international nomenclature. They were characterized according to the ISCT criteria^23^ via the expression of cell surface markers and their potential to differentiate at the single cell level into adipocytes, osteoblasts and chondrocytes as previously described^22, 24^. Their potential to differentiate into myofibroblast-like cells has been more recently reported^6^. PRP effects have also been investigated on adipose-derived stromal cell (ASCs) primary cultures. They were derived from paired chin-and knee-fat depots from a healthy of 72 year old female subject as described previously^8^. Briefly, chin- and knee-fat sampling was performed using 10-ml syringes and was centrifuged at 12,000 × *g* for 3 min. The lower liquid phase and the oily upper phase were withdrawn. Fat pads were then dissociated with 200 mg/ml collagenase and the stromal vascular fraction (SVF) was separated from the adipocyte fraction by low speed centrifugation. The SVF was then seeded on tissue culture plates and adherent cells were maintained in proliferation medium composed of DMEM (low glucose) containing 10% foetal calf serum, 10 mM HEPES, 100 U/ml penicillin and streptomycin and supplemented with 2.5 ng/ml FGF2, as previously reported^24^. ASCs were characterized by their capacity to generate adipocytes as previously described^8^. Adipose tissue samples were collected, as scraps from surgical specimens, with the informed consent of the subject, and of the baby’s parents for hMADS cells. All methods were approved and performed in accordance with the guidelines and regulations of the Review Board of the Centre Hospitalier Universitaire de Nice.

### PRP preparation and profiling

Whole blood was collected from six healthy donors and the six individual PRPs were prepared using the RegenACR-C Extra kit according to the manufacturer’s instructions (Regen Lab, Switzerland). The donors’ age and gender for the individual PRPs were: PRP A: 19 year old female; PRP B 35 year old: female; PRP C: 31 year old female; PRP D: 48 year old female; PRP F: 72 year old male and PRP G: 67 year old female. PRP was frozen for activation before the first use^25^. PRP was then diluted at the indicated doses, where 20% represent for a fivefold dilution in the culture medium in the presence of 20 μg/ml heparin. PRPs were not pooled but used individually. Blood samples were collected with the informed consent of the six blood donors. All methods were approved and performed in accordance with the guidelines and regulations of Review Board of the Centre Hospitalier Universitaire de Nice.

Simultaneous detection of multiple cytokines released from PRP was determined using the RayBio C_Series Human Growth Factor Antibody kit (RayBiotech, Inc, US). The specific antibodies on the array were for the detection of: amphiregulin (AR, AREG), bFGF, b-NGF, EGF, EGF R, FGF-4, FGF-6, FGF-7, GCSF, GDNF, GM-CSF, HB-EGF, HGF, IGFBP-1, IGFBP-2, IGFBP-3, IGFBP-4, IGFBP-6, IGF-I, IGF-I R, IGF-II, M-CSF, M-CSF R, NT-3, NT-4, PDGF R a, PDGF R b, PDGF-AA, PDGF-AB, PDGF-BB, PLGF, SCF, SCF R, TGF alpha, TGF beta 1, TGF beta2, TGF beta3,VEGF-A,VEGFR2,VEGFR3,VEGF-D. PRP profiling was performed by the Tebu-Bio service (France) according to manufacturer’s instructions.

### ELISA and collagen secretion

IGFBP3 and PDGF-AA were quantified using Sigma-Aldrich (Saint-Louis) ELISA kits and TGFβ1 using the Biosciences (London) ELISA kit. Leptin using the TECO medical ELISA kit (Sissach, Switzerland). Secretion of type I collagen in response to PRP treatment was quantified with an enzyme-linked immunosorbent assay according the supplier’s instructions (PCI C-peptide EIA KIT, Takara Bio Inc, Japan).

### Assessment of ASC differentiation

ASCs were maintained in proliferation medium untill they reached confluence. Then, cells were induced to undergo differentiation in adipogenic medium composed of DMEM supplemented with 10% FCS, 0.5 mM isobutyl-methylxanthine, 0.25 μM dexamethasone, 0.2 nM triiodothyronine, 1 μg/ml insulin and 1 μM rosiglitazone (BRL49653, a PPARγ agonist), 20 μg/ml heparin in the absence or presence of PRP.

Lipid accumulation was assessed by Oil Red O staining. Cells were stained as previously described^26^ and images were recorded under light microscopy. Then, Oil Red O stained cells were quantified by washing in water and lipid-bound Oil Red-O was dissolved in isopropanol for 15 min. Dissolved Oil Red O absorbance was determined at 490 nm on a microplate reader (BioRad iMark).

Adipogenic-and myofibroblast-gene expression was performed by quantitative PCR. RNAs were purified on RNeasy columns (Qiagen, France). RNA sample concentrations were determined using a Nanodrop spectrophotometer (Thermo Scientific, Waltham, MA, USA). Real-time PCR assays were run on a StepOnePlus system (Applied Biosystems, Life Technologies France). Transcript expression levels were evaluated using the comparative CT method (2-deltaCT). Delta delta Ct values were used when the ‘expression relative to *TBP*’ was indicated. The TATA-binding protein gene expression (*TBP*) was used for sample normalization.

Primer sequences used for real-time PCR were:

*PPARγ*: Fw: AGCCTCATGAAGAGCCTTCCA*;* Rv: TCCGGAAGAAACCCTTGCA

Adiponectin Fw: GCAGTCTGTGGTTCTGATTCCATAC; Rv: GCCCTTGAGTCGTGGTTTCC

*FABP4* Fw: GGGACGTTGACCTGGACTGA; Rv: GGGAGAAAATTACTTGCTTGCTAAA

*ColA1* Fw: ACCTGCGTGTACCCCACTCA; Rv: CCGCCATACTCGAACTGGAA

Alpha smooth muscle actin Fw: TGGATCAGCAAACAGGAATACG; Rv: GCATTTGCGGTGGACAATG

*TBP* Fw: ACGCCAGCTTCGGAGAGTTC; Rv: CAAACCGCTTGGGATTATATTCGA

For protein preparation and Western blot analysis cells were rinsed with PBS and solubilized in stop buffer containing 50 mM Hepes, at pH 7.2, 150 mM NaCl, 10 mM EDTA, 10 mM Na_4_P_2_O_7_, 2 mM Na_3_VO_4_, and 1% Triton X-100 supplemented with Protease Inhibitor Cocktail (Roche). Antibodies against Phospho-Smad2 (Ser465/467) and total Smad2/3 were from Cell Signaling. Anti-Tubulin was from Sigma and used according to the manufacturer’s instructions. Western-blots were performed as previously described^27^.

For immunofluorescence, cells were fixed for 10 min in Roti-Histofix (Roth, Lauterbourg, France). Nonspecific signals were blocked with 3% bovine serum albumin containing 0.1% tween-20 and 0.1% Triton X-100 for 30 min permeabilization. α smooth muscle actin (αSMA) antibody was from BD-Biosciences (Le Pont de Claix, France) and the secondary Alexa Fluor® 488 conjugated antibody was purchased from Life Technologies SAS (Saint Aubin, France). Cells were mounted in Mowiol containing Hoechst, and visualized with an Axiovert microscope (Carl Zeiss, Le Pecq, France) under oil immersion. Images were taken on a Zeiss Axio Observer microscope with a EC Plan Neofluar 40X (NA 1.3) oil objective using AxioVision 4.8.2 software and captured using AxioVision software (Zeiss).

### Statistical analysis

The data are the means ± SEM. Statistical analyses were performed using InStat3 software. A nonparametric unpaired test (Mann-Whitney or Student’s *t*-test) was used. P values < 0.05 were considered significant.

## Electronic supplementary material

supplementary information
