# Supplementary material for: Platelet-rich plasma respectively reduces and promotes adipogenic and myofibroblastic differentiation of human adipose-derived stromal cells via the TGFβ signalling pathway
Source: Sci Rep. 2017 Jun 7;7:2954. doi: 10.1038/s41598-017-03113-0 (PMC5462784; doi:10.1038/s41598-017-03113-0)
Supplement: Supplementary file 1 — supplementary information [file 41598_2017_3113_MOESM1_ESM.pdf]

Platelet-rich plasma respectively reduces and promotes adipogenic and myofibroblastic differentiation of human adipose-derived stromal cells via the TGFβ signalling pathway

Bérengère Chignon-Sicard[1,2], Magali Kouidhi[1], Xi Yao[1], Audrey Delerue-Audegond[3], Phi Villageois[1], Pascal Peraldi[1], Patricia Ferrari[4], Yves Rival[2], David Piwnica[3], Jérôme Aubert[3] and Christian Dani[1*]

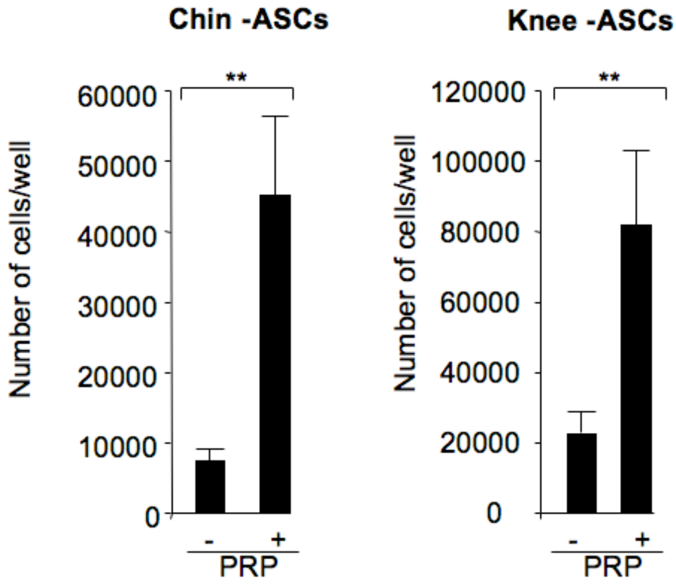

Fig. S1: Platelet-rich plasma (PRP) promotes proliferation of adipose-derived stromal cells from paired chin or knee fat sites

Adipose-derived stromal cells were isolated from paired chin and knee paired fat tissues and expanded *ex vivo*. Cells were seeded and 24h hours after cells were maintained in the absence or presence of 20% PRP. The cell number was counted after 72 h. The values were significantly different (p<0.01, n=5 individual PRP).

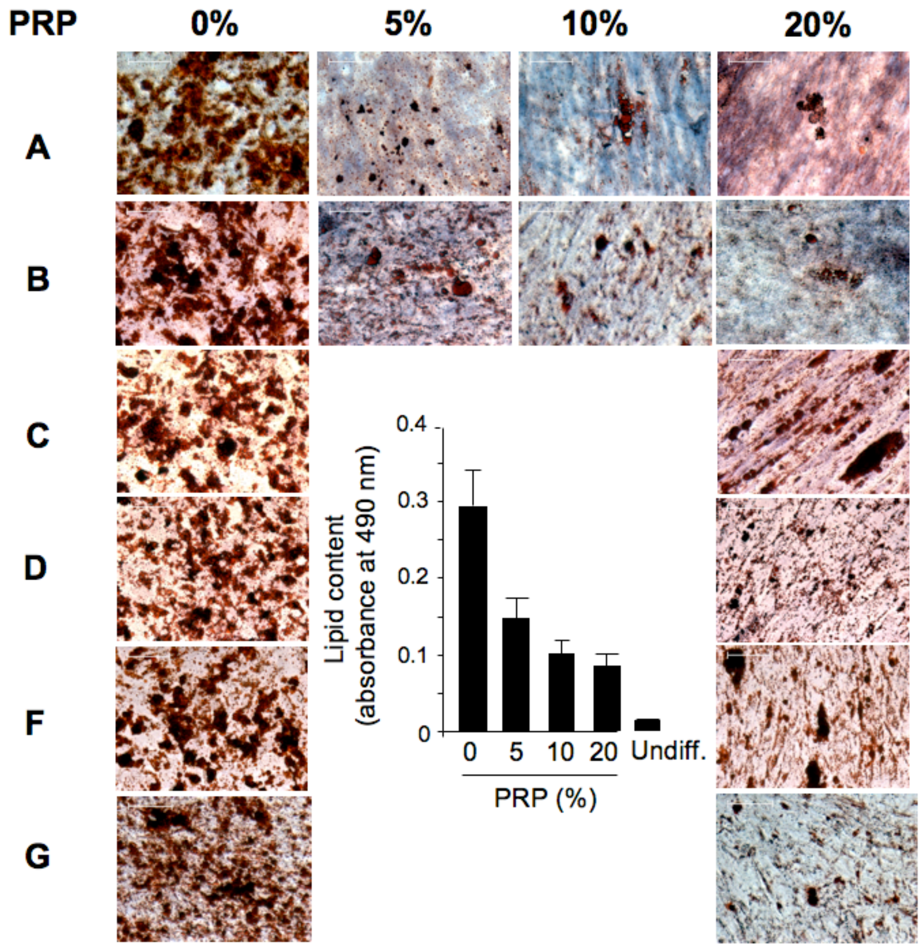

Fig. S2: Effect of individual PRPs on ASC adipocyte differentiation.

Knee-ASCs were induced to undergo adipocyte differentiation in the presence of 5%, 10% or 20% of PRP A and B; and of 20% of other PRPs. After 10 days, cells were fixed and stained with oil red-O to visualized lipid droplets. Images were recorded, then lipid content was measured by quantification of oil red-O staining. Data are the mean of 2 independent experiments (PRPA and PRP B).

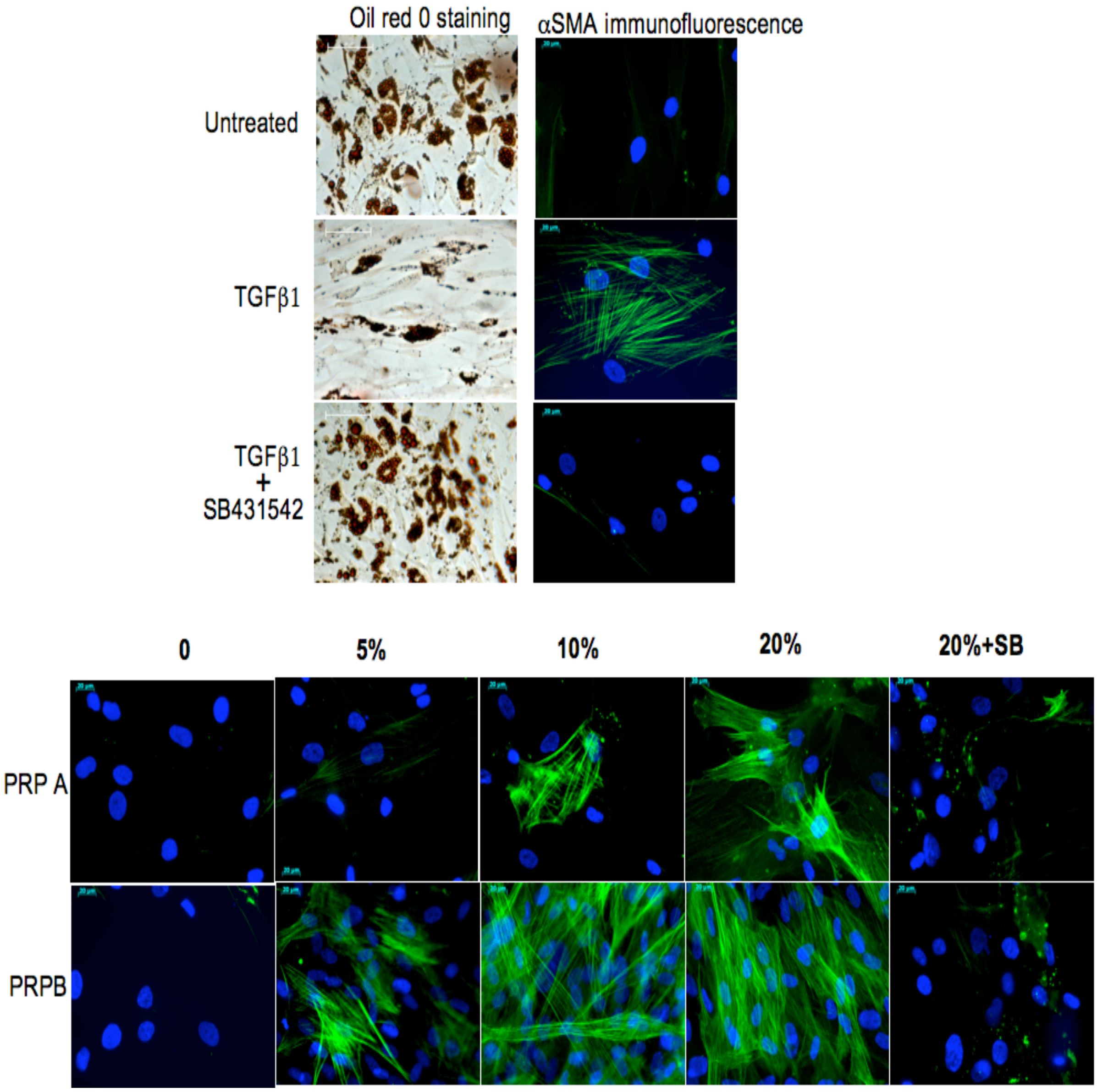

Fig. 3S :**Regulation of adipogenic and myofibroblastic differentiations by TGFb1 and PRP is reversed by SB431542**.

Upper panel: ASCs (hMADScs) were induced to undergo adipocyte differentiation in the presence of 2 ng/ml TGFβ1 in the absence or presence of 5 µM SB431542. After 10 days, cells were fixed and stained with oil red-O for adipogenic differentiation (left panel) and staining for aSMA expression by immunofluorescence for myofibroblastic differentiation (right panel).

Lower panel: ASCs (hMADScs) were induced to undergo adipocyte differentiation in the presence of different concentrations of indicated PRPs in the absence or presence of 5 µM SB431542. After 10 days cells were stained for αSMA expression by immunofluorescence.

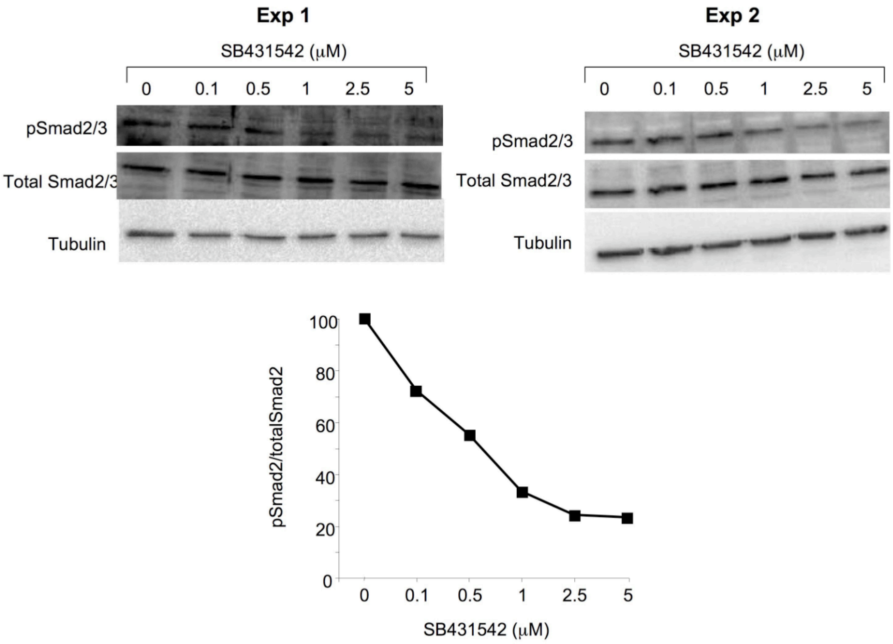

Fig.S4: Inhibition of TGFβ-induced Smad 2/3 activiation by SB431542.

ASCs were stimulation for 30 min with 5 ng/ml of TGFβ1 in the presence of different SB431542 concentrations. Then, phospho Smad2/3 was analyzed by Western-blot.

Two experiments are shown (upper part). Then, the pSmad 2/3 signals were corrected by the total Smad signals and the condition TGFb in the absence of SB431542 was taken as 100 for pSmad2/3 signal quantification (lower part).

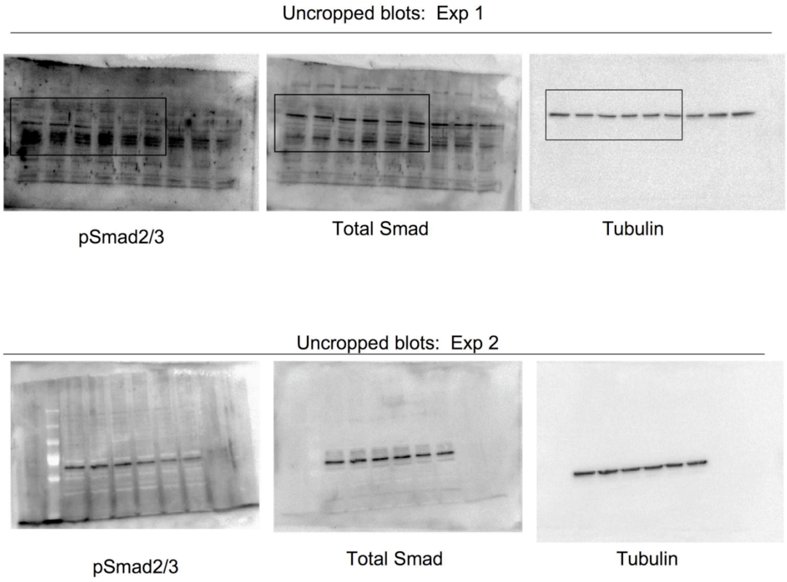

Full-length blots of Fig.S4

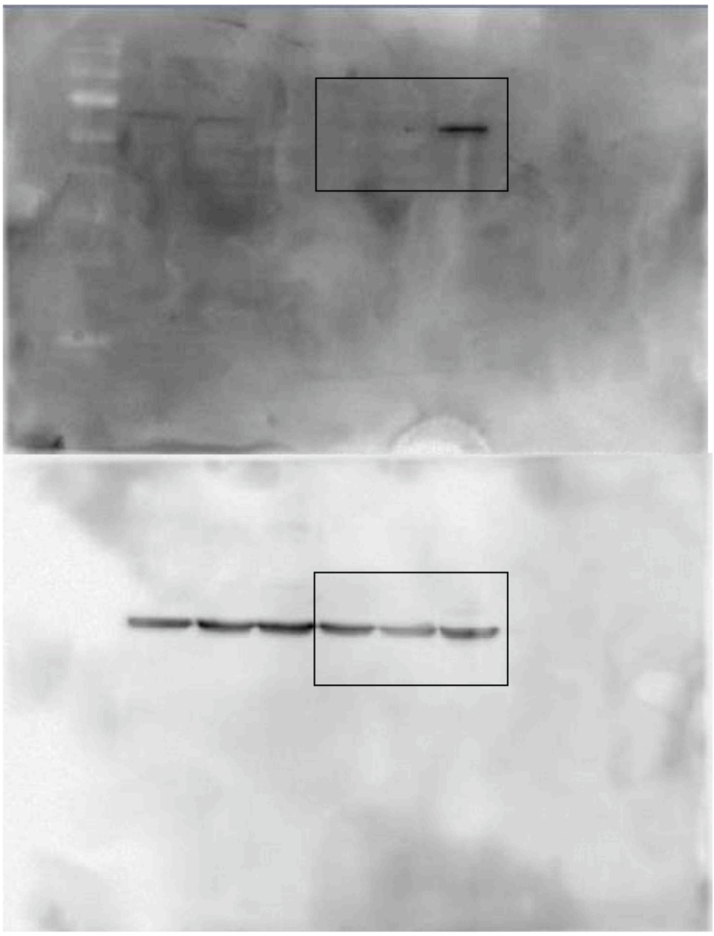

Fig. S5: Full-length blots of the main figure 3.

Factors	Concentration (ng/ml)	±SEM	Number of PRP tested
IGFBP3	1937	204	6
Leptin	9.88	2.28	6
PDGF-AA	1671	292	4
TGF β1	8.4	1.7	6

Table 1: Quantification of factors released from PRP.

IGFBP3, PDGF-AA, TGFβ1 and leptin were quantified by ELISA. The number of PRPs analyzed are indicated.
